# Developing a novel calcium silver zeolite for caries management

**DOI:** 10.1186/s12903-024-04878-3

**Published:** 2024-09-16

**Authors:** Laura Jiaxuan Li, Christie Ying-Kei Lung, Kelsey Xingyun Ge, Ke Song, Chun-Hung Chu, Ollie Yiru Yu

**Affiliations:** 1 Faculty of Dentistry, The University of Hong Kong, 34 Hospital Road, Hong Kong, S.A.R., China; 2 Department of Stomatology, Tongji Hospital, Tongji Medical College, Huazhong University of Science and Technology, Wuhan, 430030 China; 3 Department of Prosthodontics and Implantology, School of Stomatology, Tongji Medical College, Huazhong University of Science and Technology, Wuhan, 430030 China

**Keywords:** Antimicrobial, Calcium, Dental Caries, Prevention, Silver, Zeolite

## Abstract

**Objective:**

To develop a novel calcium silver zeolite (Ca-Ag-Zeo) and assess its biocompatibility, physiochemical properties and antimicrobial effects.

**Methods:**

Ca-Ag-Zeo was synthesized using ion-exchange method with calcium chloride, silver nitrate and Zeolite X (Zeo). Silver zeolite X (Ag-Zeo) and Zeo were set as control. The chemical structure, morphology, crystal structure and elemental composition of Ca-Ag-Zeo was characterized by X-ray diffraction spectrum, scanning electron microscopy, transmission electron microscopy and energy dispersive spectroscopy, respectively. Its biocompatibility on the human gingival fibroblasts was assessed by cell counting kit-8 assay. Its physiochemical properties were determined by the released calcium and silver ion using Inductive Coupled Plasma Emission Spectrometry for up to 12 weeks. The antimicrobial properties on *Streptococcus mutans*, *Lactobacillus acidophilus*, *Lactobacillus casei*, *and Candida albicans* were assessed by minimum bactericidal concentration (MBC) or minimum fungicidal concentration (MFC) assay.

**Results:**

Ca-Ag-Zeo with a hexagonal cage structure was synthesized. As for biocompatibility, the half-maximal inhibitory concentration (± SD in mg/mL) of Ca-Ag-Zeo, Ag-Zeo and Zeo in human gingival fibroblasts were 0.52 ± 0.05, 0.15 ± 0.01 and 3.35 ± 0.58, respectively (Zeo > Ca-Ag-Zeo > Ag-Zeo; *p* < 0.05). As for physiochemical properties, the accumulated ion release (± SD in mg) of Ca-Ag-Zeo, Ag-Zeo and Zeo were 0.011 ± 0.003, 0 and 0 for calcium ion, respectively (Ca-Ag-Zeo > Ag-Zeo, Zeo; *p* < 0.001), and 0.213 ± 0.032, 0.209 ± 0.019 and 0 for silver ion, respectively (Ca-Ag-Zeo, Ag-Zeo > Zeo; *p* < 0.001). As for anti-microbial ability, the MBC/MFC (mg/mL) of Ca-Ag-Zeo, Ag-Zeo and Zeo were 32, 16 and > 256 against *Streptococcus mutans*; 32, 16, > 256 against *Lactobacillus acidophilus*; 16, 16, and 256 against *Lactobacillus casei*; 0.25, 0.125; and 2, 1, > 256 against *Candida albicans*, respectively.

**Conclusion:**

A novel Ca-Ag-Zeo was developed. It presented better biocompatibility compared to Ag-Zeo. It released calcium and silver ions sustainably, and it could inhibit the growth of common cariogenic microorganisms.

## Introduction

Dental caries is prevalent worldwide affecting 90% of the world’s population [1]. It is a major health care burden and a key to global health prevention and control. Caries develops due to an imbalance between the demineralization and remineralization of the dental hard tissues [2]. The etiology of caries is complex, which mainly involved the interaction between cariogenic microorganisms, host, environment and time [3]. Pathogens metabolize sugar and produce acid on the tooth surface, causing demineralization of the dental hard tissues. Therefore, dental materials that can inhibit the growth of cariogenic microorganisms and promote remineralisation are considered a potential option for caries control.

Zeolite can be used as a carrier to adsorb antimicrobial agents, such as metal ions, and release the agents slowly over a period of time because of its porous properties. It is a crystalline microporous aluminosilicate consisting of a three-dimensional framework of [SiO_4_]^4−^ and [AlO_4_]^5−^ tetrahedron connected by shared oxygen atoms [4]. Zeolite has strong physical and chemical stability, thermal stability and biocompatibility [5], making it a promising dental material in the complex oral environment. The stability and ion exchange capacity of zeolite depend on their structure and silica to aluminum ratio. Zeolite with cage-like structures, large pores [6] and low silica-to-aluminum ratio [7] are favorable for ion exchange and can bind more metal cations stably. Zeolite can be classified according to their structure as sodalite (SOD), clinoptilolite (HEU), zeolite A (LTA), ZSM-5 (MFI), ferrite (FER), zeolite X, Y (FAU), mordenite (MOR), zeolite beta (BEA), and EMC-2(EMT). Among them, zeolite X is suitable as a carrier for caries control agents due to its large pore size and low silica to aluminum ratio [8].

However, zeolite has very limited antimicrobial and no remineralizing effects [9]. A solution to enhance its capabilities for caries control is to load metal ions into the zeolite frameworks. Zeolite compounds for dental applications are mostly combined with metals or metal derivatives. Metals such as silver, zinc, calcium, strontium and their derivatives can be integrated into zeolite for dental applications [9]. Metallic zeolite that have been used in caries management are silver zeolite [10], zinc zeolite [11] and calcium zeolite [12]. Silver zeolite and zinc zeolite have antimicrobial properties that can inhibit the attachment and growth of microorganisms on dental hard tissues [13]. However, silver zeolite and zinc zeolite cannot repair hard tissues that have already been demineralized [14]. Calcium zeolite, which may promote remineralization of dental hard tissue, were seldom used for dental application. However, calcium is much less antimicrobial than silver, which is a broad-spectrum antimicrobial agent.

Therefore, this study was conducted to develop and characterize a novel calcium silver zeolite (Ca-Ag-Zeo) with antimicrobial and remineralizing properties for caries management and determine its biocompatibility, calcium and silver ion release kinetics and antimicrobial effects against common cariogenic microorganisms.

## Materials and methods

### Development of calcium silver zeolite and silver zeolite

#### Synthesis of calcium silver zeolite (Ca-Ag-Zeo)

This study used faujasite zeolite in sodium molecular sieves 13X (sodium zeolite X) (Sigma, Aldrich, Germany) and calcium chloride (Sigma, Aldrich, Germany) to synthesis calcium zeolite (Ca-Zeo) for further synthesis. Calcium was loaded into zeolite by an ion exchange method. A 0.1 M calcium chloride (CaCl_2_) solution was prepared. 2 g of sodium zeolite was mixed with 40 mL of 0.1 M CaCl_2_ solution with stirring at 80ºC for 24 h. The Ca-Zeo were separated by centrifugation (5000 rpm) and dried in an oven at 120˚C overnight [15]. The Ca-Ag-Zeo was synthesized by loading silver into Ca-Zeo. A 2 g of Ca-Zeo was mixed with 40 mL of 0.1 M silver nitrate (Sigma, Aldrich, Germany) solution at room temperature. The suspension was stirred in the dark condition and heated to 70˚C for 6 h [16]. The Ca-Ag-Zeo were separated by centrifugation at 5000 rpm and dried in an oven at 60˚C for overnight [17]. The collected Ca-Ag-Zeo were ground and stored in a brown bottle.

#### Synthesis of silver zeolite (Ag-Zeo) as control

Ag-Zeo were prepared as a control group. 1 g of sodium zeolite X was added in 20 mL of 0.1 M silver nitrate solution at room temperature to form a suspension. The suspension was stirred in the dark condition at 70 °C for 6 h. The silver zeolite was separated by centrifugation at 5000 rpm and dried at 60 °C for 6 h. The collected Ag-Zeo were ground and stored in a brown bottle.

#### Group information

All the assessment were performed on three types of zeolite compounds, Group Ca-Ag-Zeo: calcium silver zeolite X; Group Ag-Zeo: silver zeolite X and Group Zeo: sodium zeolite X. Group Ca-Ag-Zeo were experimental group, while Groups Ag-Zeo and Zeo were set as control. The group information was shown in Table 1.

**Table 1:** The group information of this research

Group	Experimental Group	Control Group	Control Group
Full name	Calcium Silver Zeolite X	Silver Zeolite X	Sodium Zeolite X
Abbreviation	Ca-Ag-Zeo	Ag-Zeo	Zeo

### Characterization of zeolite compounds

#### Surface morphology and elemental composition

Zeolite compounds’ surface morphology was assessed by Scanning Electron Microscope with a voltage of 5 kV (SEM, Leo 1530 FEG Scanning Electron Microscope; Leo, Oberkochen, Germany). Before analysis, the samples were coated with a fine carbon layer using a BAL-TEC SCD004 sputter coating system in order to improve the electrical conductivity. Silver is photosensitive. Exposure to light causes silver to be photo-oxidized, and in the process free radicals are produced, which can attack the metal and destroy its structure [18]. To avoid silver oxidation under light conditions, Ag-Zeo and Ca-Ag-Zeo powders were stored away from light prior to experimentation. Energy-dispersive X-ray spectroscopy (EDS) under the SEM was used to analyze zeolite compounds’ elemental composition with a voltage of 15 kV. The particle diameter of zeolite was measured by the ruler included in the SmartSEM system.

#### Microstructure and crystal structure

The microstructure of zeolite compounds was assessed by transmission electron microscopy (TEM, Thermo Scientific Talos F200X STEM; Thermo, Massachusetts, United States). The samples were characterized using powder and the solution of 5 mg zeolite compounds powder dissolved in 0.5 ml DI water was dropped on a clean carbon film. The particle diameter of zeolite was measured by the ruler included in the TEM system.

The crystal structure of zeolite compounds was characterized by selective area electron diffraction (SAED) using high-resolution transmission electron microscopy (HRTEM, FEI Tecnai G2 20 Scanning TEM; FEI, Oregon, the United States). After the suspension was dropped on a clean carbon film, the lattice streaks of zeolite compounds were studied by HRTEM. The HRTEM operates at 200 kV and is equipped with a slow scan cooled CCD (Gatan) camera and digtalmicrograph software.

#### Chemical structure

The chemical structure of synthesized powder samples was determined by X-ray diffraction (XRD, Philips PW1710 diffractometer; Philips, Amsterdam, Netherlands) with CuKα radiation (λ = 1.5406 Å) operated at 45 kV and 30 mA. The diffraction patterns were collected in the 2θ range of 5–80° at 0.25°/min with a step size of 0.02°.

### Biocompatibility

This study was approved by the Institutional Review Board of the University of Hong Kong (IRB No. UW 22–554). The human gingival fibroblasts (HGFs) were purchased from ATCC HK as HGF-1 CRL-2014 ™. HGFs were grown in DMEM supplemented with 10% fetal bovine serum (FBS), 100 U/mL penicillin, and 100 µg/mL streptomycin were subcultured (1 × 10^4^ cells/mL) in 96-well plates (100µL per well). The cell lines were grown at 37 °C in a humidified environment containing 5% carbon dioxide for 24 h in DMEM for cell attachment. When the cells were completely attached to the plates, the medium was removed. Medium containing pre-weighed Ca-Ag-Zeo, Ag-Zeo and Zeo powder were added to the well at different concentrations (0.3125-8 mg/mL) for 24 h, and culture medium as the control. To measure cytotoxicity, cells were placed in DMEM containing 0.1 g/mL Cell Counting Kit-8 and incubated at 37 °C for 2 h, then the absorbance at 450 nm was measured using a microplate reader. To evaluate cytotoxicity, relative cell viability was calculated, and the half-maximal inhibitory concentration (IC50) of the three zeolite compounds on the cells was calculated, which is the amount of chemical required to inhibit cell viability by 50%. The relative cell viability of zeolites was calculated by [19]:

$$\begin{gathered} Cell\,viability\% = \hfill \\\frac{{OD\,value\,(Test) - OD\,value\,(Blank)}}{{OD\,value\,(Control) - OD\,value\,(Blank)}} \times 100\% \hfill \\ \end{gathered}$$

3 samples per group were assessed in each assessment of biocompatibility and the assessment was replicated for 3 times (*n* = 9).

### The calcium and silver ion release kinetics

The silver and calcium ion release were assessed using a charge-coupled device detector inductively coupled plasma optical emission spectroscopy (ICP-OES). 0.32 g of powder of each specimen was dissolved in 5 ml of deionized water to achieve a concentration of 64 mg/ml. The specimen were stored in room temperature until time for measurement. The solution were centrifuged at 5,000 rpm for 5 min before each measurement. After centrifugation, the liquid will be divided into two parts, the supernatant and the precipitate. The supernatant was aspirated with the pipette and the concentration of calcium and silver ions in the supernatant was measured by ICP. To detect the amount of newly released ions per day/week, the remaining precipitate was redissolved in 5 ml of DI water and mixed with the tube shaker (IKA Shakers Vortex 1) and left to stand until the next measurement. Studies have shown that ion release from zeolite compounds is usually fast at the beginning of the experiment and gradually slows down with time [20]. The measurements were conducted daily for the first week and then weekly until Week 12. 8 samples in each group (*n* = 8) were assessed and the average was taken as the result of calcium and silver ion release.

### Antimicrobial properties

Minimum bactericidal concentration (MBC) or minimum fungicidal concentration (MFC) tests were conducted to evaluate the antimicrobial effects of the zeolite compounds. The MBC assessment were performed on *Streptococcus mutans*, *Lactobacillus acidophilus*, *Lactobacillus casei*, and while the MFC were conducted on *Candida albicans* with a serial two-fold dilution of solutions containing zeolite compounds from 256 to 0.125 mg/mL.

10 µL bacterial culture (10^6^ CFU/mL) in brain heart infusion (BHI) broth was added to each well of 96-well plates containing 100µL serial two-fold dilutions of solutions containing zeolite compounds. The solution was cultured anaerobically for 24 h. 5 µL of fluid was pipetted from each well and incubated in blood agar at 37 °C for 48 h. 3 samples per group were assessed in each assessment of antimicrobial and the biological replicate of the test was conducted for 3 times (*n* = 9).

10 µL fungi culture (10^6^ CFU/mL) in Sabouraud Dextrose Agar (SDA) was added to each well of 96-well plates containing 100µL serial two-fold dilutions of experimental solutions. The solution was cultured anaerobically for 24 h. 5 µL of fluid was pipetted from each well and incubated in and Sabouraud dextrose agar at 37 °C for 48 h. 3 samples per group were assessed in each assessment of antimicrobial and the biological replicate of the test was conducted for 3 times (*n* = 9).

### Data analysis

The quality data were analysed using SPSS Statistics 20 (IBM Corporation, Somers, NY, USA) and Origin 2021 (OriginLab Corporation, Northampton, USA). The cumulative concentration value of calcium and silver ions released were analyzed by One-way Analysis of Variance, Tukey’s test and Tamhane’s T^2^ test. The significance level was set at 95%.

## Results

### Preparation and characterization of Ca-Ag-Zeo

#### Surface morphology and elemental composition of zeolite compounds

**Fig. 1:**
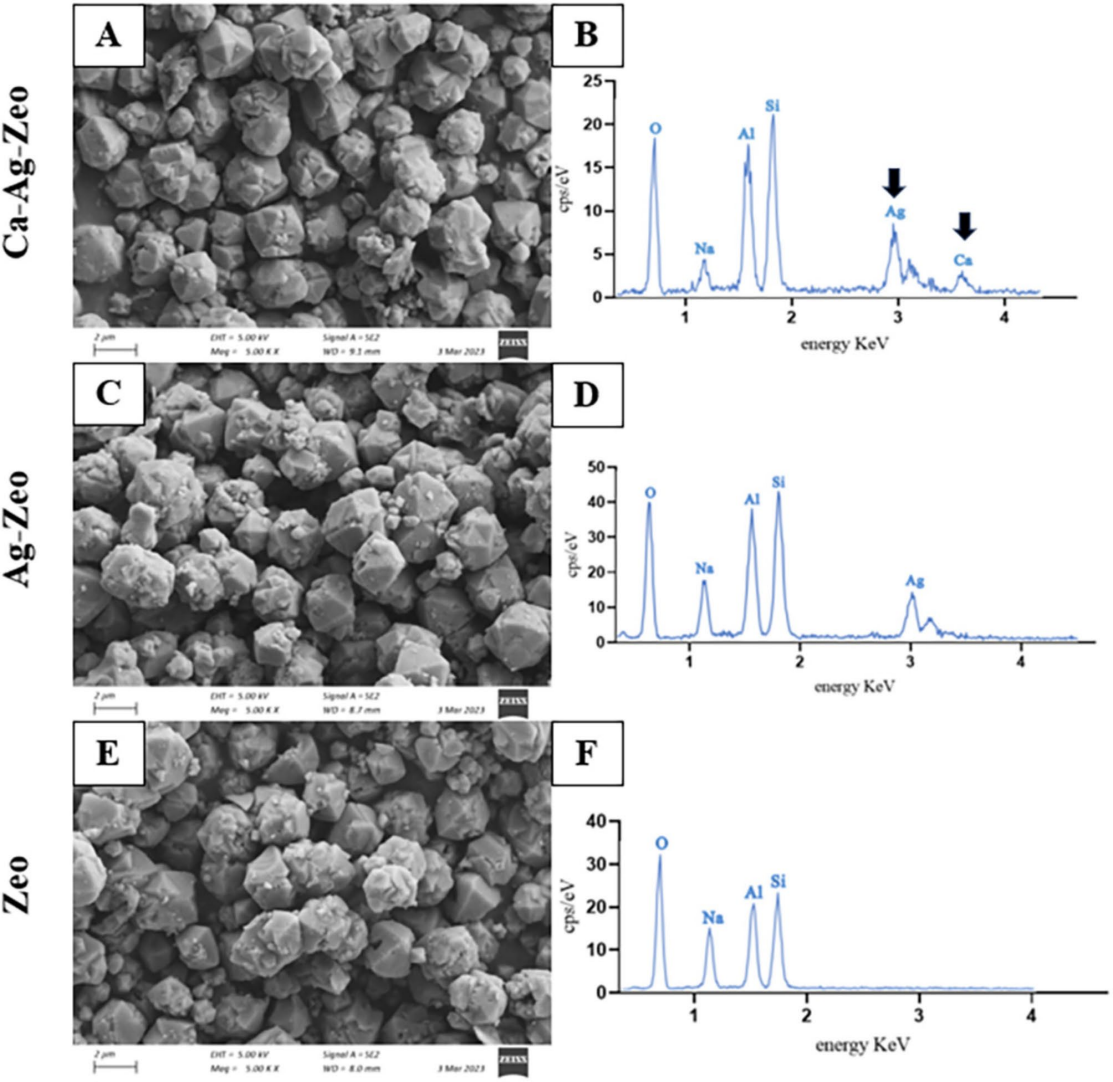
The scanning electron microscope images of Ca-Ag-Zeo (**A**), Ag-Zeo (**C**) and Zeo (**E**); EDS images of Ca-Ag-Zeo (**B**), Ag-Zeo (**D**) and Zeo (**F**). *Ca-Ag-Zeo*, *calcium silver zeolite; Ag-Zeo*, *silver zeolite; Zeo*, *zeolite. Si*, *silicon; Al*, *aluminum; Ag*, *silver; Ca*, *calcium; Na*, *sodium; O*, *oxygen*

The scanning electron microscope (SEM) micrographs of zeolite compounds were shown in Fig. 1 (A, C,E). The zeolite compounds in this study had micron particles with uniform particle size. The average particle sizes of Ca-Ag-Zeo, Ag-Zeo and Zeo were in the range of 1.5–3.0 μm. As shown in Fig. 1, Ca-Ag-Zeo and Ag-Zeo had almost the same shape characteristics as Zeo, and both retain the typical properties of FAU zeolites, with octahedral morphology, cubic structure, and sharp edges. It indicated that the exchange of calcium and silver ions did not affect the surface morphology of zeolite particles.

**Fig. 2:**
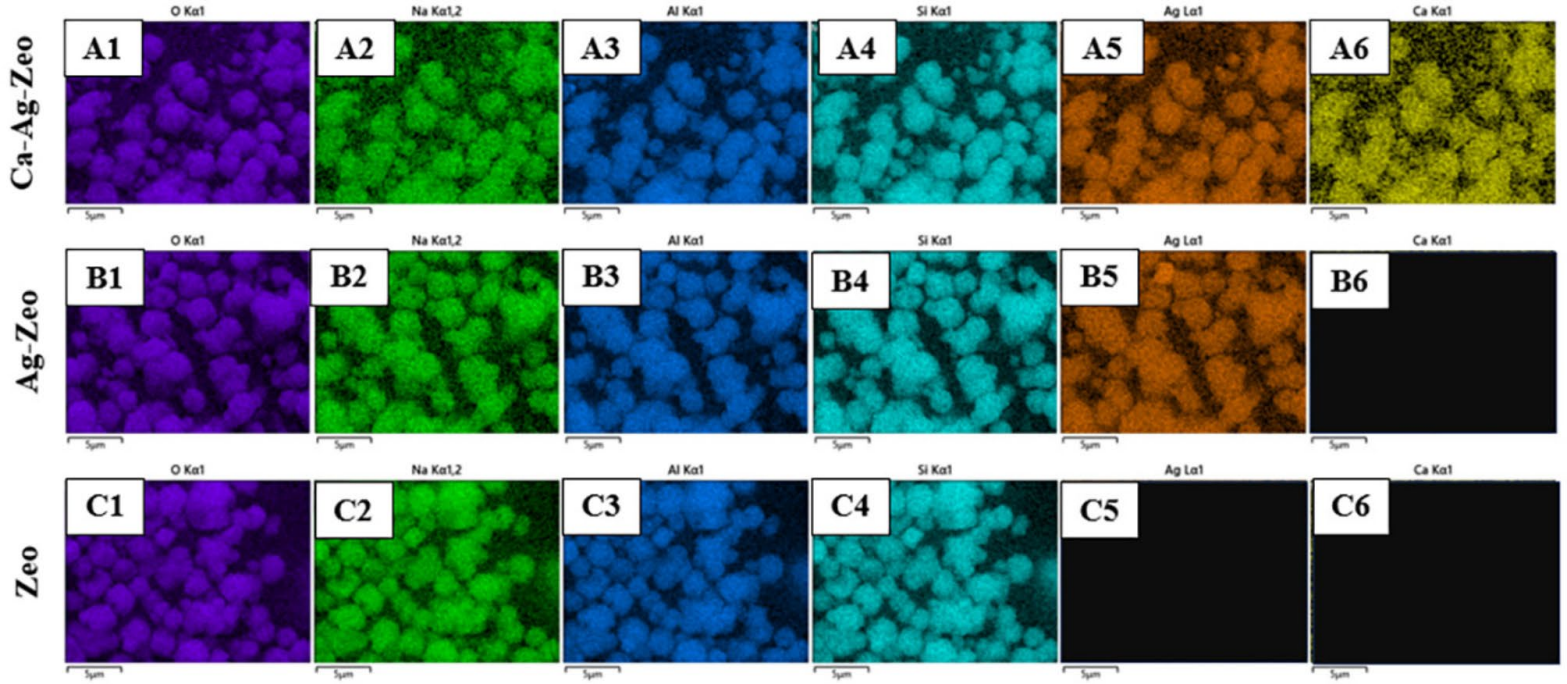
The Energy-dispersive X-ray spectroscopy element mapping images of Ca-Ag-Zeo (A1-A6), Ag–Zeo (B1-B6) and Zeo (C1-C6). *Ca-Ag-Zeo*, *calcium silver zeolite; Ag-Zeo*, *silver zeolite; Zeo*, *zeolite*

The Energy-dispersive X-ray spectroscopy (EDS) analysis of the zeolite samples was shown in Fig. 1 (B, D,F). The EDS analysis revealed that the major elements of the zeolite X used in the experiment were oxygen, sodium, aluminum and silicon. The EDS spectrum of Ca-Ag-Zeo showed a peak at 3.0 KeV and a peak at 3.7 KeV, confirming the presence of silver and calcium, respectively. The EDS spectrum proved the successful synthesis of Ca-Ag-Zeo and Ag-Zeo. The EDS elemental mapping of available elements in the sample was shown in Fig. 2. The Ca-Ag-Zeo mapping diagram showed that the calcium and silver elements were distributed in all zeolite particles homogeneously. The percentage of elemental composition of Ca-Ag-Zeo, Ag-Zeo and Zeo was shown in Table 2.

**Table 2:** Elemental composition percentage (Wt%) of Ca-Ag-Zeo, Ag–Zeo and zeo

Element (Wt%)	O	Na	Al	Si	Ag	Ca	Total
Ca-Ag-Zeo	39.21	7.04	15.23	19.03	16.68	2.81	100.00
Ag-Zeo	40.51	8.17	15.22	19.03	17.07	0.00	100.00
Zeo	43.53	13.56	18.53	24.38	0.00	0.00	100.00

*Ca-Ag-Zeo*, *calcium silver zeolite; Ag-Zeo*, *silver zeolite; Zeo*, *zeolite*

#### Crystal structure of zeolite compounds

**Fig. 3:**
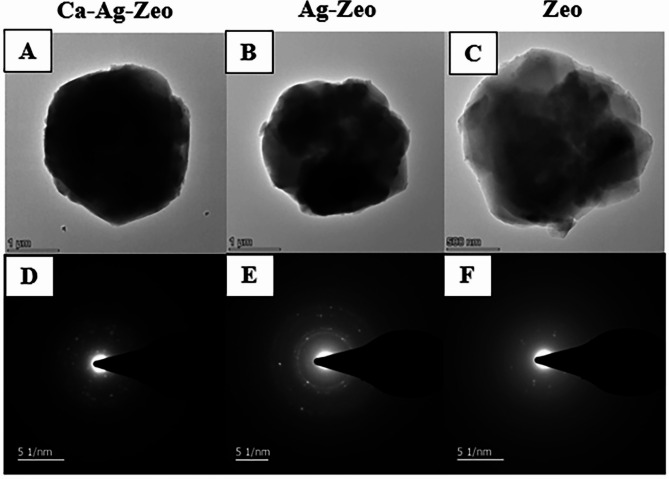
TEM images of Ca-Ag-Zeo (**A**), Ag–Zeo (**B**) and Zeo (**C**) and SAED pattern of Ca-Ag-Zeo (**D**), Ag–Zeo (**E**) and Zeo (**F**). *Ca-Ag-Zeo*, *calcium silver zeolite; Ag-Zeo*, *silver zeolite; Zeo*, *zeolite*

The transmission electron microscopy (TEM) images of the zeolite samples were shown in Fig. 3 (A, B,C). The TEM images showed that Ca-Ag-Zeo, Ag-Zeo and Zeo had cubic structures with particle sizes in the range of 1.5–3.0 μm and sharp crystal surfaces, which was consistent with the SEM results. The diffraction ring diameters of Ca-Ag-Zeo, Ag-Zeo and Zeo were consistent as shown in selected area electron diffraction (SAED) Fig. 3 (D, E,F), indicating that the presence of silver and calcium ions did not cause crystalline change in the particle structure of zeolite X.

#### Chemical structure of zeolite compounds

**Fig. 4:**
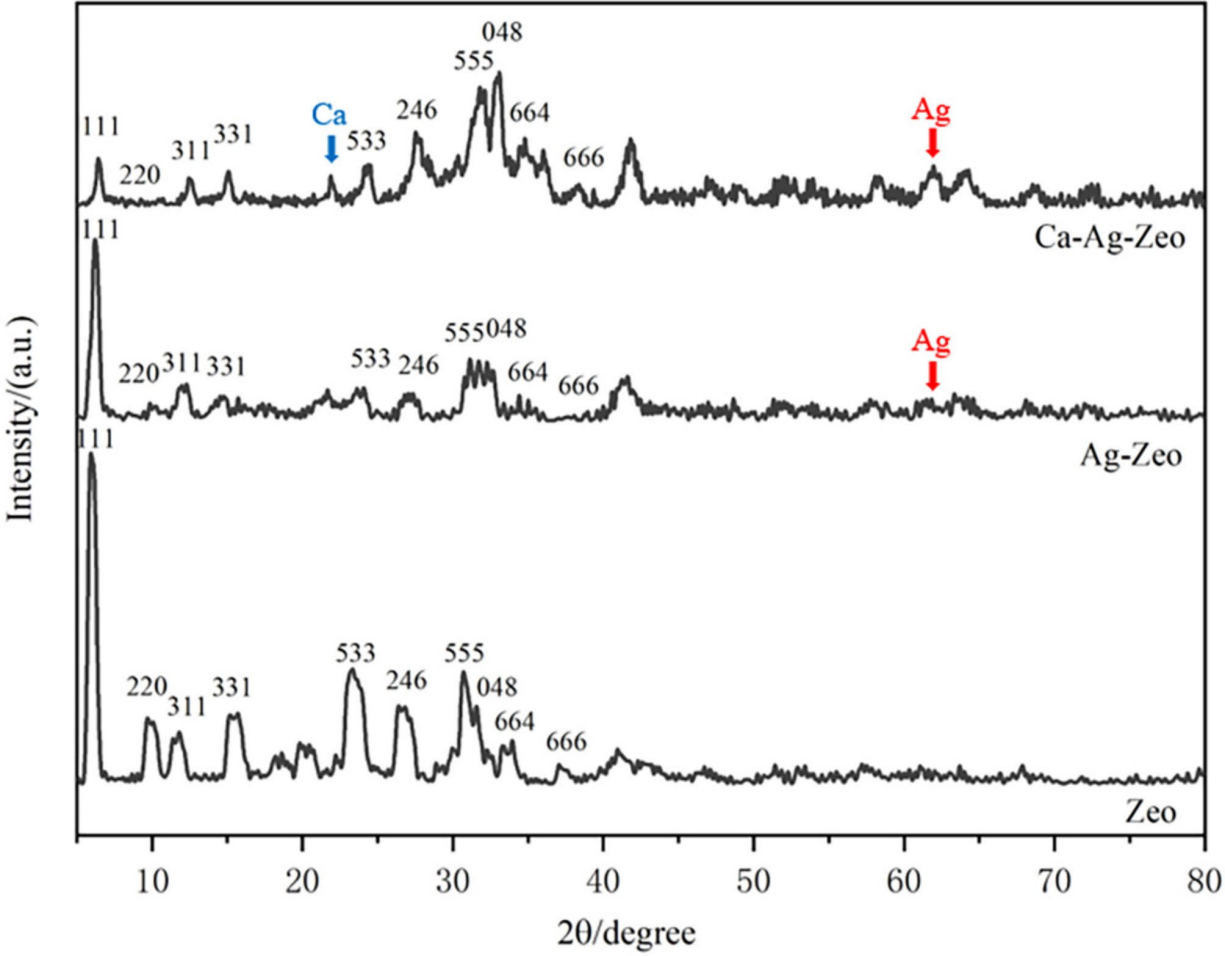
X-ray diffractograms of Ca-Ag-Zeo, Ag-Zeo and Zeo, the number represents the indices of crystal face of the zeolite crystal. *Ca-Ag-Zeo*, *calcium silver zeolite; Ag-Zeo*, *silver zeolite; Zeo*, *zeolite*

The X-ray diffractograms (XRD) of the zeolite samples were shown in Fig. 4. The XRD patterns of Ca-Ag-Zeo, Ag-Zeo and Zeo all exhibited series of characteristic peaks at 2θ = 6.2°(111), 9.98°(220), 11.73°(311), 15.43°(331), 23.28°(533), 26.65°(246), 30.94°(555), 31.98°(048) 33.59°(664) and 37.34°(666), which matched well with the standard FAU zeolite X [21], indicating that the addition of calcium and silver did not affect the crystal structure of zeolite. In addition, diffraction peaks appeared at 2θ = 64.5° and 2θ = 21.7° in the spectrum of Ca-Ag-Zeo, indicating the presence of silver [22] and calcium [23], respectively. The peak appeared at 2θ = 64.5° representing silver were found Ag-Zeo, while no characteristic peaks of calcium and silver were found in Zeo.

### Biocompatibility

**Fig. 5:**
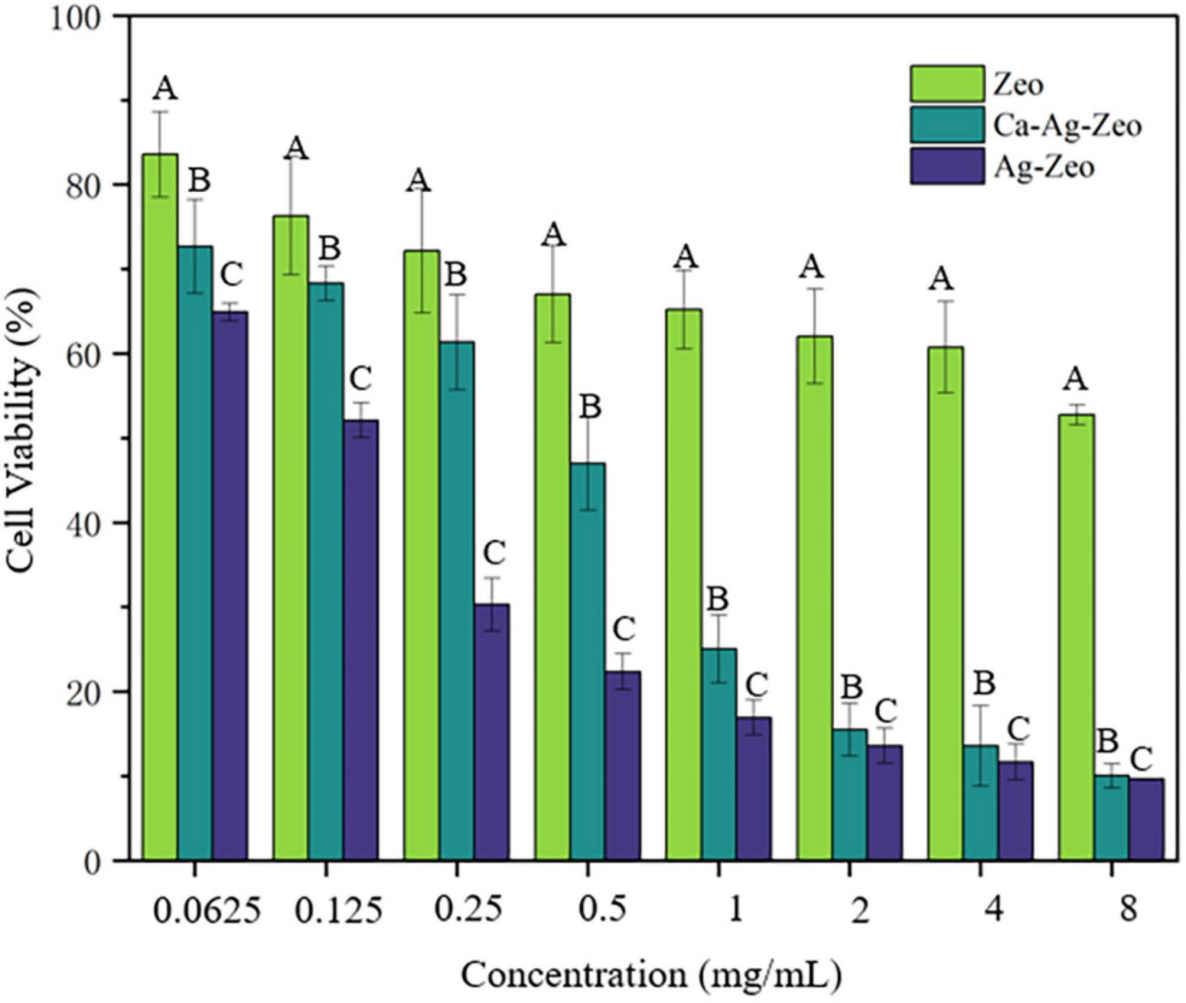
Cell viability of human gingival fibroblasts treated with 0.0625, 0.125, 0.25, 0.5, 1, 2, 4, and 8 mg/mL of Ca-Ag-Zeo, Ag-Zeo, and Zeo for 24 h. For each unit of measurement, different capital letters indicate statistical differences after Tukey’s test (*p* < 0.05). A represent the cell viability of Zeo; B represent the cell viability of Ca-Ag-Zeo; C represent the cell viability of Ag-Zeo. A > B > C (*p* < 0.05).*Ca-Ag-Zeo*, *calcium silver zeolite; Ag-Zeo*, *silver zeolite; Zeo*, *zeolite*

The biocompatibility of zeolite compounds on human gingival fibroblasts (HGFs) were determined by the OD values and IC50 value (Fig. 5). The IC50 value of Ca-Ag-Zeo, Ag-Zeo and Zeo were 0.52 ± 0.05 mg/mL, 0.15 ± 0.01 mg/mL and 3.35 ± 0.58 mg/mL, respectively (Ag-Zeo < Ca-Ag-Zeo < Zeo, *p* < 0.05). The IC50 value of Ag-Zeo was lower than Zeo (*p* < 0.05). The IC50 value of Ca-Ag-Zeo was higher than Ag-Zeo (*p* < 0.05).

### The calcium and silver ion release kinetics

**Fig. 6:**
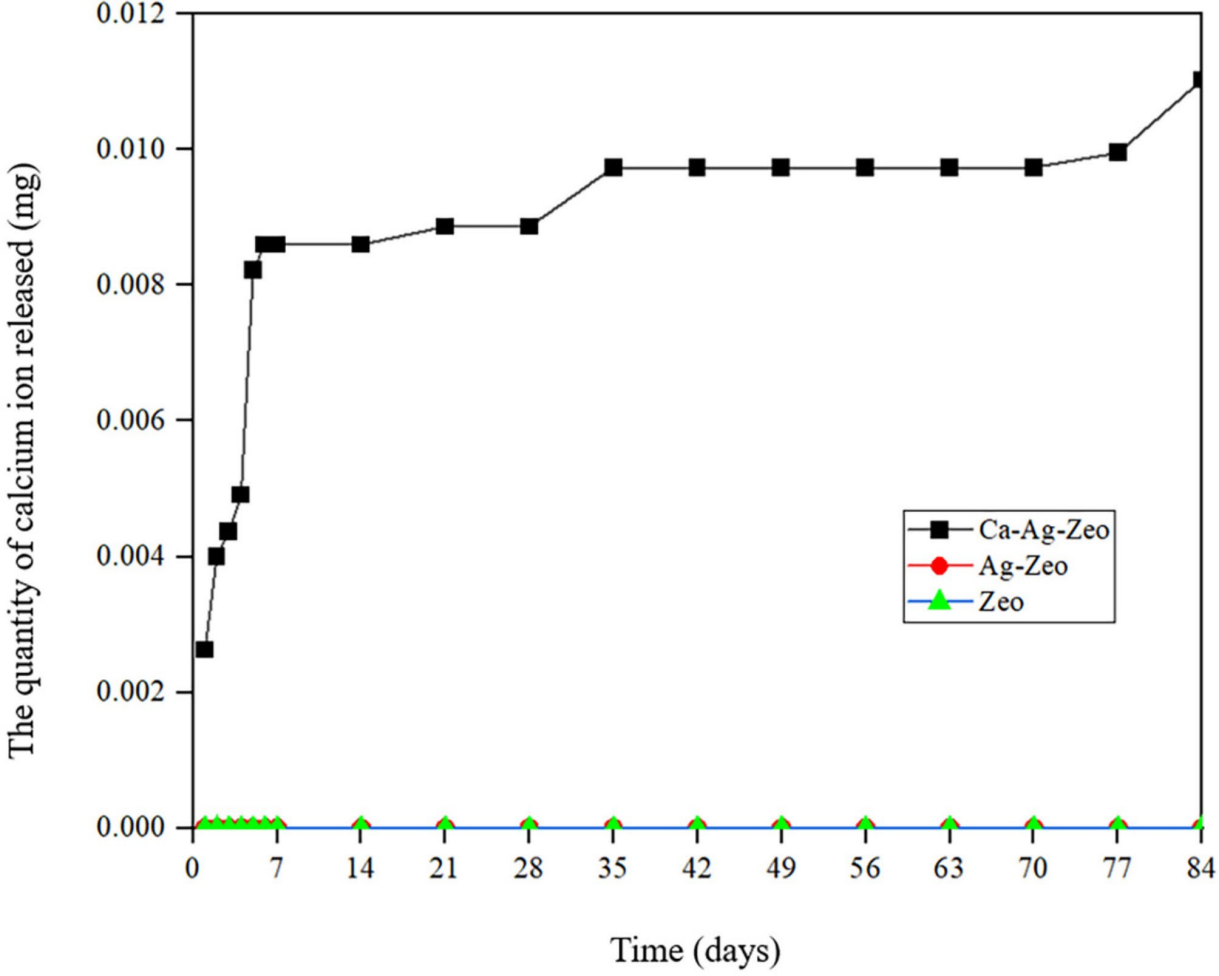
Accumulated amount of calcium released from Ca-Ag-Zeo, Ag-Zeo and Zeo powder over 84 days. *Ca-Ag-Zeo*, *calcium silver zeolite; Ag-Zeo*, *silver zeolite; Zeo*, *zeolite*

The results in Fig. 6 showed that the release of calcium ions from Ca-Ag-Zeo increased rapidly during the first week. Calcium ion release slowed down after one week. At the end of the study at week 12, calcium ion was detectable from the Ca-Ag-Zeo group, with the average cumulative release of calcium ions reaching 0.011 ± 0.003 mg. Ag-Zeo and Zeo did not release calcium ions for 12 weeks.

**Fig. 7:**
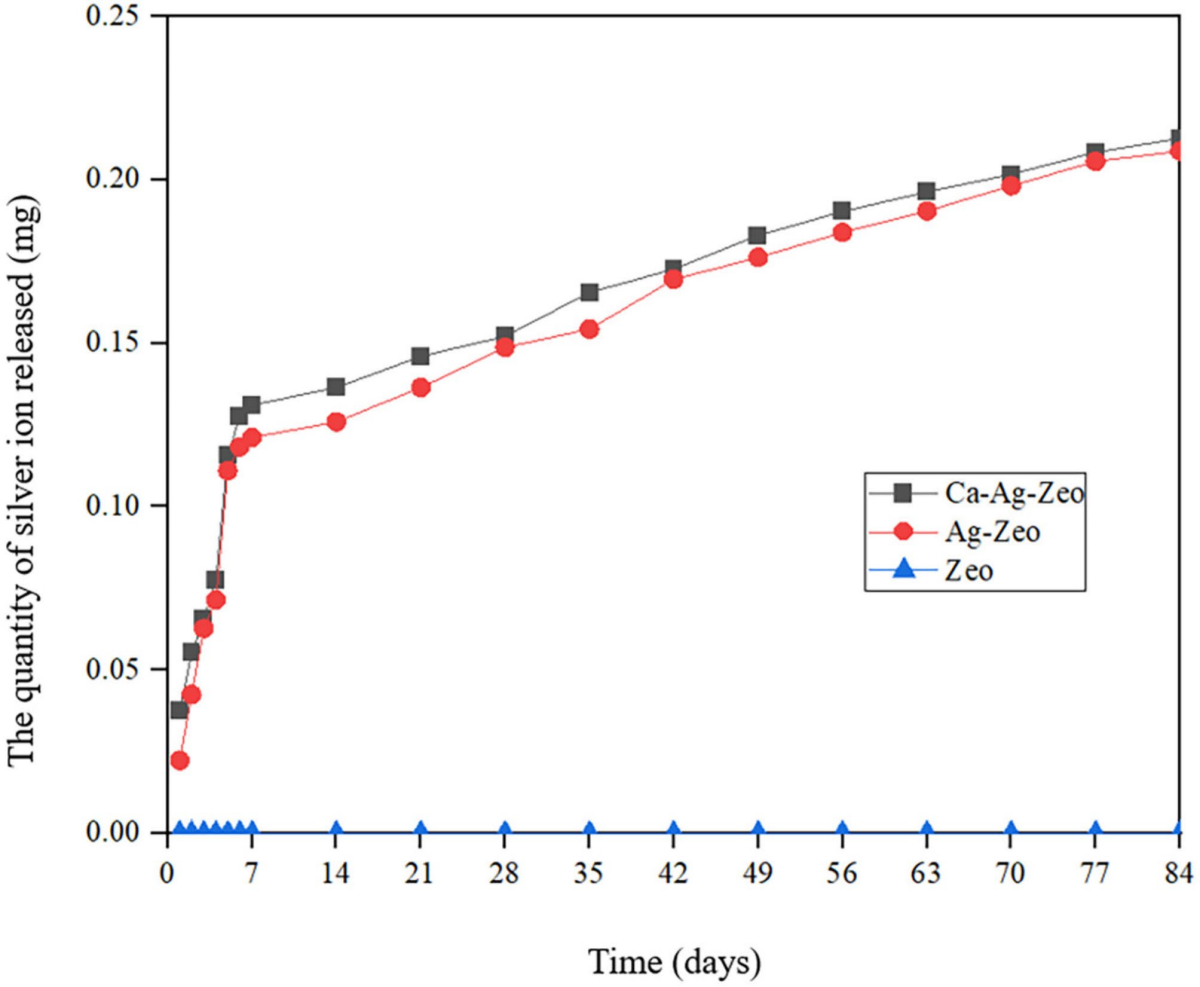
Accumulated amount of silver released from Ca-Ag-Zeo, Ag-Zeo and Zeo powder over 84 days. *Ca-Ag-Zeo*, *calcium silver zeolite; Ag-Zeo*, *silver zeolite; Zeo*, *zeolite*

Figure 7 showed the cumulative silver ion release from Ca-Ag-Zeo, Ag-Zeo and Zeo over 12 weeks. Ca-Ag-Zeo released silver ions steadily over a 12-week period. Ca-Ag-Zeo and Ag-Zeo also released silver ions at similar rates, and the rate of silver ion release was higher during in the first week compared to the latter stage. No statistical difference were found between the cumulative silver ion release amount of Ca-Ag-Zeo (0.213 ± 0.032 mg) and Ag-Zeo (0.209 ± 0.019 mg) over the 12-week period (*p* > 0.05). Zeo did not release silver ions.

### Antimicrobial properties

The MBC/MFC values of Ca-Ag-Zeo, Ag-Zeo and Zeo against *S. mutans*, *L. acidophilus*, *L. casei* and *C. albicans* were shown in Table 3. The inhibitory effect of Ca-Ag-Zeo on the growth of common oral pathogenic microorganisms was similar to that of Ag-Zeo and much higher than that of Zeo. MBC/MFC values of Ca-Ag-Zeo on *S. mutans*, *L. acidophilus*, and *C. albicans* were slightly higher than those of Ag-Zeo (Table 2). For *L. casei*, the MBC values were 16 mg/mL for both Ca-Ag-Zeo and Ag-Zeo (Table 2).

**Table 3:** Minimum bactericidal concentration (MBC) and Minimum Fungicidal Concentration (MFC) of Ca-Ag-Zeo, Ag-Zeo and zeo

Cariogenic Microorganism	MBC/MFC of Ca-Ag-Zeo (mg/mL)	MBC/MFC of Ag-Zeo (mg/mL)	MBC/MFC of Zeo (mg/mL)
*S. mutans*	32	16	> 256
*L. acidophilus*	32	16	> 256
*L. casei*	16	16	> 256
*C. albicans*	2	1	> 256

*Ca-Ag-Zeo*, *calcium silver zeolite; Ag-Zeo*, *silver zeolite; Zeo*, *zeolite*

## Discussion

This study has developed a novel calcium silver zeolite (Ca-Ag-Zeo). The Ca-Ag-Zeo released calcium and silver ions sustainably over a period of 12 weeks. Ca-Ag-Zeo had strong inhibitory effects on common cariogenic microorganism, including *S. mutans*, *L. acidophilus*, *L. casei*, and *C. albicans*. Ca-Ag-Zeo had antimicrobial properties to inhibit plaque. It also could provide calcium ions to promote remineralization of demineralized hard tissues. Therefore, it has great potential for caries management applications.

Zeolite X was used to synthesize Ca-Ag-Zeo in this study. Zeolite is considered to be a promising cation carrier, which allows for the slow exchange of cations in the pores of its framework [24]. Zeolite can exchange water, ions and polar molecules with its surroundings [25] and can bind with metal ions to exert antimicrobial activity. This important property depends mainly on the structural porosity and pore size [26]. The most commonly used zeolite in the oral cavity are LTA zeolite (zeolite A) and FAU zeolite (zeolite X and zeolite Y). Zeolite A have a pore size from three angstroms to five angstroms (3–5 Å) [27], whereas the zeolite 13X, the zeolite X used in our study, has an pore size of eight angstroms (8 Å) [28]. Since zeolite X has larger pore channels than zeolite A, the ion exchange capacity of zeolite X is higher than that of zeolite A. Our pilot study confirmed that silver zeolite X has a stronger antimicrobial effect than silver zeolite A. Therefore, we used silver zeolite X to synthesized Ca-Ag-Zeo instead of using a commercial silver zeolite product, which used zeolite A as a framework for silver incorporation. In addition, Zeolite X has a silica-to-aluminum ratio between 1.1 and 1.5, while Zeolite Y has a silica-to-aluminum ratio greater than 1.5. The lower the silica-to-aluminum ratio of a zeolite, the easier it is for it to exchange with cations with a high charge density. Zeolite X has better ion exchange property than Zeolite Y. Therefore, Zeolite X were used to synthesize Ca-Ag-Zeo in this study.

Ca-Ag-Zeo presented particles of 1.5–3 μm diameter, octahedral morphology with a cubic crystal structure, which was consistent with the morphology of zeolite X reported in previous studies [29]. Ca-Ag-Zeo was prepared by exchanging the calcium and silver ions with the original sodium ions in the zeolite. The aluminosilicate framework of zeolite consisted mainly of a tetrahedral three-dimensional framework composed of silicon, aluminum, and oxygen [4]. Exchangeable cations that balance the negative charge of the alumino-silicate framework were present in the windows and cavities of the zeolite, so that the exchange of cations did not affect the main structure of the framework [30].

The XRD reflection peaks of Ca-Ag-Zeo and zeolite X were almost identical, indicating that the Ca-Ag-Zeo we synthesized retained the FAU structure. This is in agreement with the results reported in the literature [29, 31, 32]. However, the intensities of the FAU peaks of Ca-Ag-Zeo and Ag-Zeo were lower than those of Zeo, indicating that the addition of calcium and silver may reduce the crystallinity of FAU zeolite. Previous studies have shown that the change in peak intensity could be due to the inhomogeneity of crystal size [33] caused by changes in atomic positions or atomic density within the crystal cell [34]. In the ion-exchange reaction of Ca-Ag-Zeo synthesis, silver and calcium ions replace sodium atoms in the lattice, which might result in a change in atomic positions in the zeolite crystals.

Ca-Ag-Zeo showed superior biocompatibility compared to Ag-Zeo in this study. Previous studies have shown that Ag-Zeo is biocompatible and non-cytotoxic [35, 36]. The lower cytotoxicity of Ca-Ag-Zeo compared to Ag-Zeo might be due to the replacement of silver by a portion of the calcium in the zeolite framework. Because silver ions showed higher toxicity compared to calcium ions, the replacement might reduce the toxicity and enhance the biocompatibility of Ca-Ag-Zeo.

Ca-Ag-Zeo released calcium and silver ions sustainably over a 12-week period. The release kinetic curves for calcium and silver ions fit best with the pseudo-second-order (PSO) kinetic model [20, 37], as evidenced by the fact that calcium and silver ions were released at higher rates during the first week than after one week. The release of silver ions from Ca-Ag-Zeo was not significantly different from that of Ag-Zeo. This indicated that the addition of calcium did not affect the release of silver ions from Ag-Zeo. In the faujasite structure of zeolite X, there are three cation exchange sites, which are located in the hexagonal prisms (site I), the sodalite cages (sites I ' and II ‘), and the supercages (sites II, III, and III ‘) [38]. Calcium ions usually bind to site I [30]. Silver ions bind to all sites except site III [39]. Moreover, due to the different adhesion sites of silver ion and calcium ion on the structure of zeolite, the addition of calcium ion does not have a significant effect on the release of silver ion.

The inhibitory ability of Ca-Ag-Zeo against *S. mutans*, *L. acidophilus*, *and L. casei* and *C. albicans* were assessed in this study. *S. mutans* is a acidogenic and aciduric bacteria, acting as an initiator of dental caries [40]. *L. acidophilus* and *L. casei* are major contributors to the progression of dental caries [41, 42]. They produces lactic acid as the main end-product of carbohydrate fermentation, which further acidify the oral environment [43]. *C. albicans* is also closely related to caries development. It has synergistic effect with *S mutans* in promoting the development of dental caries by promoting the adhesion and acid production of *S mutans* [44]. It also produces collagenase which can degrade the organic component of dental hard tissues [45]. In this study, Ca-Ag-Zeo inhibit the growth of these cariogenic microorganisms, indicating its potential in the application of caries prevention. The antimicrobial effect of Ca-Ag-Zeo is mainly dependent on the amount and rate of silver ion release. Previous studies have suggested four possible modes of inhibitory action of silver ions: (i) disruption of bacterial cell membranes by silver ions and their dissolution; (ii) inhibition of cellular replication by silver ions by binding to and denaturing bacterial DNA and RNA; (iii) generation of reactive oxygen species (ROS) and inhibition of intracellular respiratory enzymes; and (iv) modulation of signal transduction pathways [46].

The silver and calcium ion release kinetics of Ca-Ag-Zeo were evaluated and the results indicated that Ca-Ag-Zeo has the ability to release ions. The released silver ions from Ca-Ag-Zeo may indicate its potential effects in preventing the growth of cariogenic biofilms. The released calcium ions may act as the ingredient of dental hard tissue and promote the remineralisation. However, the anti-biofilm and the remineralising effects of the Ca-Ag-Zeo have not been assessed in this study. Further studies should be conducted to confirm its anti-caries effects.

The limitation of this study is that there is no direct experimental evidence on the caries preventive effectiveness and remineralization ability of Ca-Ag-Zeo on caries demineralized areas. Whether the calcium ions released by Ca-Ag-Zeo can play a significant and positive role in clinical prevention and treatment needs to be further investigated by more clinical trials.

In the future, Ca-Ag-Zeo with its antimicrobial properties and remineralization potential could be used in dental adhesives and restorative materials to prevent secondary caries and prolong the life of restorations in caries management. In addition, this novel material can be used for apical surgery to prevent apical infections; periodontal surgery to treat periodontitis; and denture restorations to prevent Candida stomatitis. In conclusion, Ca-Ag-Zeo is a prospective dental material, which will be promising in the fields of Cariology, Endodontics, Periodontics, and Prosthodontics.

## Conclusion

A novel calcium-silver zeolite with good biocompatibility has been developed. It can continuously release calcium and silver ions and can inhibit the growth of common cariogenic microorganisms. It has great potential in caries prevention and treatment.

## Data Availability

All data generated or analyzed during this study are included in this published article.

## Abbreviations

Ag-Zeo: Silver zeolite X
BHI: Brain heart infusion
*C. albicans*: *Candida albicans*
Ca-Ag-Zeo: Calcium silver zeolite
CaCl_2_: Calcium chloride
Ca-Zeo: Calcium zeolite
EDS: Energy-dispersive X-ray spectroscopy
FBS: Fetal bovine serum
HGFs: Human gingival fibroblasts
IC50: Half-maximal inhibitory concentration
ICP-OES: Inductively coupled plasma optical emission spectroscopy
*L. acidophilus*: *Lactobacillus acidophilus*
*L. casei*: *Lactobacillus casei*
MBC: Minimum bactericidal concentration
MBC: Minimum bactericidal concentration
MFC: Minimum fungicidal concentration
MFC: Minimum fungicidal concentration
PSO: Pseudo-second-order
ROS: Reactive oxygen species
*S. mutans*: *Streptococcus mutans*
SAED: Selective area electron diffraction
SDA: Sabouraud Dextrose Agar
SEM: Scanning Electron Microscope
TEM: Transmission electron microscopy
XRD: X-ray diffraction
Zeo: Zeolite X
